# Supplementary material for: Single Nucleotide Polymorphisms May Increase the Risk of Aspiration Pneumonia in Post-Stroke Patients with Dysphagia
Source: Curr Issues Mol Biol. 2022 Aug 19;44(8):3735–45. doi: 10.3390/cimb44080255 (PMC9406641; doi:10.3390/cimb44080255)
Supplement: Supplementary file 1 [file cimb-44-00255-s001.zip › cimb-1844745-supplementary.pdf]

Single Nucleotide Polymorphisms May Increase the Risk of Aspiration Pneumonia in Post-Stroke Patients with Dysphagia

Hae-Yeon Park [1], Hyun-Mi Oh [2,3], Tae-Woo Kim [2,3], Youngkook Kim [4], Geun-Young Park [5], Hyemi Hwang [5], and Sun Im [5,*]

*Correspondence to Sun Im, MD, PhD.
Department of Rehabilitation Medicine, Bucheon St. Mary's Hospital, College of Medicine, The Catholic University of Korea, 327, Sosa-ro, Bucheon-si, Gyeonggi-do, 14647, Republic of Korea.
Tel: +82-32-340-2170, E-mail: lafoliamd@gmail.com, lafolia@catholic.ac.kr

Supplementary Table S1. Baseline parameters according to APoE genotypes in different age groups

	Age <65			Age≥65		
	**APoE ε4 (−) (n=81)**	**APoE ε4 (+) (n=22)**	**P value**	**APoE ε4 (−) (n=77)**	**APoE ε4 (+) (n=26)**	**P value**
Sex (male)	49 (60.5)	20 (90.9)	0.015*	46 (59.7)	21 (80.8)	0.088
BMI	22.7±2.8	22.9±3.5	0.797	22.3±3.7	21.4±3.0	0.285
Stroke type			0.191			0.708
infarction	32 (39.5)	13 (59.1)		56 (72.7)	18 (69.2)	
hemorrhage	45 (55.6)	9 (40.9)		20 (26.0)	7 (26.9)	
both	4 (4.9)	0 (0.0)		1 (1.3)	1 (3.8)	
Location			0.400			0.654
supratentorial	57 (70.4)	16 (72.7)		58 (75.3)	18 (69.2)	
infratentorial	18 (22.2)	6 (27.3)		18 (23.4)	7 (26.9)	
multiple	6 (7.4)	0 (0.0)		1 (1.3)	1 (3.8)	
Side			0.503			0.877
right	28 (34.6)	5 (22.7)		29 (37.7)	11 (42.3)	
left	34 (42.0)	12 (54.5)		37 (48.1)	11 (42.3)	
bilateral	19 (23.5)	5 (22.7)		11 (14.3)	4 (15.4)	
DM	30 (37.0)	7 (31.8)	0.840	38 (49.4)	10 (38.5)	0.462
HBP	57 (70.4)	12 (54.5)	0.253	59 (76.6)	21 (80.8)	0.868
NIHSS	12.0 [7.0-19.0]	15.0 [10.0-20.0]	0.420	13.0 [7.0-18.0]	14.0 [5.0-18.0]	0.796
Intubation	37 (45.7)	10 (45.5)	1.000	25 (32.5)	10 (38.5)	0.750
Tracheostomy	20 (24.7)	8 (36.4)	0.412	14 (18.2)	4 (15.4)	0.979
Alcohol (yes)	26 (32.1)	11 (50.0)	0.193	22 (28.6)	9 (34.6)	0.739
Smoking (yes)	21 (25.9)	11 (50.0)	0.057	21 (27.3)	9 (34.6)	0.643
MMSE	22.0 [9.0-28.0]	17.0 [6.0-26.0]	0.353	16.0 [6.0-24.0]	17.5 [12.0-24.0]	0.760
BBS	19.0 [2.0-47.0]	13.0 [0.0-38.0]	0.451	4.0 [1.0-30.0]	3.0 [0.0-28.0]	0.468
NPM at ≥12weeks	27 (33.3)	10 (45.5)	0.424	35 (45.5)	8 (30.8)	0.279
Aspiration pneumonia	30 (37.0)	16 (72.7)	0.006*	48 (62.3)	15 (57.7)	0.851
MBSImp-Oral	11.0 [6.0-16.0]	11.5 [7.0-18.0]	0.486	11.0 [8.5-15.0]	10.5 [9.0-16.0]	0.938
MBSImp-Pharyngeal	9.0 [5.0-13.0]	11.0 [9.0-13.0]	0.065	9.0 [6.0-13.5]	8.5 [5.0-12.0]	0.300
GUSS	4.0 [1.0-14.0]	3.0 [1.0-4.0]	0.052	3.0 [1.0- 5.0]	3.5 [1.0-5.0]	0.670
PAS	8.0 [6.0-8.0]	8.0 [8.0-8.0]	0.029*	8.0 [8.0- 8.0]	8.0 [7.0- 8.0]	0.538

MASA	141.0 [104.0-172.0]	122.5 [107.0-141.0]	0.178	139.0 [101.0-154.0]	141.5 [119.0-160.0]	0.549
EAT-10	40.0 [28.0-40.0]	40.0 [38.0-40.0]	0.680	40.0 [32.0-40.0]	40.0 [40.0-40.0]	0.166
FOIS	1.0 [1.0-1.0]	1.0 [1.0-1.0]	0.079	1.0 [1.0-1.0]	1.0 [1.0-1.0]	0.704
FAC	0.0 [0.0-3.0]	0.0 [0.0-2.0]	0.373	0.0 [0.0-2.0]	0.0 [0.0- 2.0]	0.696
MBI	39.0 [3.0-64.0]	16.0 [0.0-64.0]	0.195	19.0 [3.0-55.0]	9.5 [3.0-65.0]	0.681
mRS (≥3)	79 (97.5)	22 (100.0)	0.459	75 (97.4)	24 (92.3)	0.565

Values are given as number (), means±SD, or median [interquartile range]. Chi-Squared test, student t-test, or Mann-Whitney test was performed to compare between groups, and P-value < 0.05 are used for statistical significance.

Abbreviations: BMI, Body mass index; Afib, Atrial fibrillation; DM, Diabetes mellitus; HBP, Hypertension; NIHSS, National Institutes of Health Stroke Scale; MMSE, Mini-mental state examination; BBS, berg balance scale; NPM, Nil per mouth; MBSImp, Modified Barium Swallow Impairment Profile; GUSS, Gugging swallowing screen; PAS, Penetration-aspiration scale; MASA, Mann Assessment of Swallowing Ability; EAT-10, Eating Assessment Tool; FOIS, Functional Oral Intake Scale; FAC, Functional ambulatory category; MBI, Modified Barthel Index; mRS, modified Rankin Scale.

Supplementary Table S2. Association between APoE genotypes and clinical outcomes of the patients categorized by age groups

	Age <65			Age≥65		
	**APoE ε4 (−) (n=81)**	**APoE ε4 (+) (n=22)**	**P value**	**APoE ε4 (−) (n=77)**	**APoE ε4 (+) (n=26)**	**P value**
NPM at ≥12weeks	27 (33.3)	10 (45.5)	0.424	35 (45.5)	8 (30.8)	0.279
FOIS			0.828			0.132
Baseline	1.0 [1.0-1.0]	1.0 [1.0-1.0]		1.0 [1.0-1.0]	1.0 [1.0-1.0]	
3 month	4.0 [2.0-6.0]	4.0 [1.0-5.0]		3.0 [1.0- 5.0]	4.0 [2.0- 5.0]	
MASA			0.699			0.579
Baseline	141.0 [104.0-172.0]	122.5 [107.0-141.0]		139.0 [101.0-154.0]	141.5 [119.0-160.0]	
3 month	172.0 [135.0-184.0]	159.5 [120.0-185.0]		156.0 [128.0-177.0]	169.0 [142.0-184.0]	
MBSImp-Oral			0.689			0.119
Baseline	11.0 [6.0-16.0]	11.5 [7.0-18.0]		11.0 [8.5-15.0]	10.5 [9.0-16.0]	
3 month	4.0 [2.0-10.0]	6.0 [3.0-10.0]		8.0 [3.0-11.0]	6.0 [2.0- 9.0]	
MBSImp-Pharyngeal			0.573			0.809
Baseline	9.0 [5.0-13.0]	11.0 [9.0-13.0]		9.0 [6.0-13.5]	8.5 [5.0-12.0]	
3 month	5.0 [1.0-9.0]	6.5 [4.0-10.0]		6.0 [3.0-10.0]	5.0 [3.0- 6.0]	
GUSS			0.586			0.178
Baseline	4.0 [1.0-14.0]	3.0 [1.0-4.0]		3.0 [1.0- 5.0]	3.5 [1.0-5.0]	
3 month	16.0 [4.0-18.0]	14.0 [2.0-17.0]		11.0 [3.0-17.0]	14.0 [4.0-17.0]	
PAS			0.490			0.431
Baseline	8.0 [6.0-8.0]	8.0 [8.0-8.0]		8.0 [8.0- 8.0]	8.0 [7.0- 8.0]	
3 month	5.0 [2.0-8.0]	6.0 [4.0-7.0]		7.0 [5.0- 8.0]	6.0 [3.0- 8.0]	
EAT-10			0.572			0.484
Baseline	40.0 [28.0-40.0]	40.0 [38.0-40.0]		40.0 [32.0-40.0]	40.0 [40.0-40.0]	
3 month	24.0 [9.0-40.0]	25.5 [4.0-40.0]		30.0 [12.0-40.0]	28.5 [9.0-40.0]	
FAC			0.313			0.532
Baseline	0.0 [0.0-3.0]	0.0 [0.0-2.0]		0.0 [0.0-2.0]	0.0 [0.0- 2.0]	
3 month	2.0 [0.0-4.0]	1.5 [0.0-4.0]		0.0 [0.0- 3.0]	0.0 [0.0- 2.0]	
MBI			0.483			0.936
Baseline	39.0 [3.0-64.0]	16.0 [0.0-64.0]		19.0 [3.0-55.0]	9.5 [3.0-65.0]	
3 month	60.0 [12.0-88.0]	61.5 [5.0-72.0]		32.0 [3.0-72.0]	36.5 [4.0-69.0]	

Values are given as number () or median [interquartile range]. Chi-Squared test or Mann-Whitney test was performed to compare between groups, and P-value < 0.05 are used for statistical significance.

Abbreviations: NPM, Nil per mouth; FOIS, Functional Oral Intake Scale; MASA, Mann Assessment of Swallowing Ability; MBSImp, Modified Barium Swallow Impairment Profile; GUSS, Gugging swallowing screen; PAS, Penetration-aspiration scale; EAT-10, Eating Assessment Tool; FAC, Functional ambulatory category; MBI, Modified Barthel Index.
